# Identification and analysis of pancreatic intraepithelial neoplasia: opportunities and challenges

**DOI:** 10.3389/fendo.2024.1401829

**Published:** 2025-01-07

**Authors:** Ling-ling Pian, Mei-hui Song, Teng-fei Wang, Ling Qi, Tie-li Peng, Ke-ping Xie

**Affiliations:** ^1^ School of Medicine, The South China University of Technology, Guangzhou, Guangdong, China; ^2^ Division of Gastroenterology, Institute of Digestive Disease, Affiliated Qingyuan Hospital, The Sixth Clinical Medical School, Guangzhou Medical University, Qingyuan People’s Hospital, Qingyuan, Guangdong, China; ^3^ Shandong Laboratory of Yantai Drug Discovery, Bohai Rim Advanced Research Institute for Drug Discovery, Yantai, Shandong, China

**Keywords:** PanIN, PDAC, KRAS, cellular origin, genetic susceptibility, diagnosis

## Abstract

Pancreatic intraepithelial neoplasia (PanIN) is the most common precursor lesion of pancreatic ductal adenocarcinoma (PDAC), which has poor prognosis with a short median overall survival of 6-12 months and a low 5-year survival rate of approximately 3%. It is crucial to remove PanIN lesions to prevent the development of invasive PDAC, as PDAC spreads rapidly outside the pancreas. This review aims to provide the latest knowledge on PanIN risk, pathology, cellular origin, genetic susceptibility, and diagnosis, while identifying research gaps that require further investigation in this understudied area of precancerous lesions. PanINs are classified into PanIN 1, PanIN 2, and PanIN 3, with PanIN 3 having the highest likelihood of developing into invasive PDAC. Differentiating between PanIN 2 and PanIN 3 is clinically significant. Genetic alterations found in PDAC are also present in PanIN and increase with the grade of PanIN. Imaging methods alone are insufficient for distinguishing PanIN, necessitating the use of genetic and molecular tests for identification. In addition, metabolomics technologies and miRNAs are playing an increasingly important role in the field of cancer diagnosis, offering more possibilities for efficient identification of PanIN. Although detecting and stratifying the risk of PanIN poses challenges, the combined utilization of imaging, genetics, and metabolomics holds promise for improving patient survival in this field.

## 1 Introduction

Pancreatic ductal adenocarcinoma (PDAC) is predicted to become the second leading cause of death in North America and Europe within the next ten years. This is mainly due to the lack of early diagnosis, effective treatment options, and the increased burden of cancer (1–4). Unfortunately, 85% of patients with PDAC are diagnosed as advanced tumors with concomitant metastases due to a lack of symptoms and early biomarkers. Although the median survival of patients with advanced PDAC has increased from 6 months to 12 months, the 5-year survival rate remains less than 3% (5–7). To reduce the mortality rate of patients with PDAC, early diagnostic biomarkers need to be discovered and early diagnosis realized.

The current significant consensus is that PDAC develops from precursor lesions known as pancreatic intraepithelial neoplasias (PanINs). In 2016, Voltaggio et al. analyzed intraepithelial neoplasia in multiple anatomical sites from risk factors and pathobiology; she described three primary intraepithelial neoplasia, all of which are non-invasive (8). Intraductal papillary mucinous neoplasms (IPMNs) and mucinous cystic neoplasms (MCNs) are macroscopic lesions, while PanINs are microscopic lesions characterized as flat or papillary, non-invasive epithelial tumors. The amount of mucus varies, and the degree of cytological and structural atypia varies as well. PanINs are classified into low-grade, moderate-grade, and high-grade lesions based on the degree of structural and cytological dysplasia (9). High-grade dysplasia is most likely to develop into invasive carcinoma (10). Additionally, the genes mutated in PanIN lesions are identical to those altered in PDAC, which helps to establish that PanIN can indeed develop into invasive cancer. Therefore, studying the progression of PanIN is crucial to early diagnose and treat of PDAC.

Telomere shortening and activating point mutations in the KRAS gene are among the earliest genetic alterations observed in PanIN. KRAS alterations hold promise as a biomarker for precursor lesions, but cannot provide information on the grade of the lesion (11, 12). Inactivating mutations in the p16/CDKN2A gene begin to appear in PanINs with low to moderate dysplasia, while inactivating mutations in the oncogenes SMAD4 and TP53 occur only in PanINs with high-grade dysplasia, indicating that they are “late” events (9, 13). Moreover, miR-21 and miR-155 have been found to be overexpressed in PanINs compared to normal pancreatic ductal cells (14). The identification and treatment of PanINs may be effective in preventing the progression of fatal PDAC (15).

## 2 Pathology of PanIN

PanIN is a common lesion found in the pancreas. In the past, due to the lack of strict diagnostic criteria, leading to various descriptions such as ductal hyperplasia, metaplasia, proliferation, dysplasia, neoplasia, and carcinoma *in situ* lesions, which have increased the difficulty of correct understanding of PanIN (16–18). However, current knowledge about PanIN is much clearer and more precise.

PanIN originates in the pancreas’s small ducts and is a microscopic neoplastic lesion in the smaller pancreatic ducts, usually less than 5 mm in diameter, and typically associated with centrilobular atrophy of the pancreatic lobules. Currently, morphological grading of PanINs is performed based on the degree of cytoarchitecture and nuclear heterogeneity and is classified into three types, PanIN 1, PanIN 2, and PanIN 3. PanIN 1 lesions are typically flat or papillary, with columnar epithelium and basally oriented round nuclei, and are rich in mucin. PanIN 2 is papillary with dysplastic nuclei, such as loss of polarity, nuclear crowding, cell enlargement, and hyperpigmentation. PanIN 3 is papillary, micropapillary, or cribriform, and possesses many features of invasive carcinomas, such as severe nuclear atypia, a general loss of nuclear polarity, significant epithelial outgrowth into the lumen, significantly reduced or absent mucin, and the presence of dystrophic cupped cells (19). Although PanIN 3 has been considered a ductal carcinoma *in situ*, it is still a non-invasive lesion bounded by the basement membrane.

While some researchers have suggested that PanIN is only a reactive chemosis lesion, the presence of cancer-associated gene mutations in most PanIN lesions indicates that PanIN is a neoplastic lesion. Similar to PDAC, the prevalence of PanINs also increases with age (20). In a study by Andea et al., the frequency and clinical characteristics of PanIN in PDAC and benign pancreas were compared. The results showed that the frequency of PanIN lesions was 82% in patients with PDAC, which was significantly higher than the 54% found in benign pancreata. Additionally, there was a progressive increase in the frequency of overall PanIN lesions (16%, 60%, and 82%, respectively) and PanIN 3 (0%, 4%, and 40%, respectively) from normal pancreas to pancreatitis and PDAC (21). These findings indirectly support the hypothesis that PanIN lesions have a precancerous role. PanIN 1 is more commonly found in benign pancreas, PanIN 2 is more frequently observed in pancreatic tumor tissue, and PanIN 3 is almost exclusively detected in PDAC (21).

A recent study of PanIN lesions in 173 significant autopsy cases without PDAC or IPMN revealed that the number of PanIN 3 lesions was positively correlated with PanIN 1 or PanIN 2. PanIN 3 is a multifocal lesion that primarily involves interlobular and intralobular ducts, along with higher-grade extralobular fibrosis. PanIN 3 is more likely to occur in diabetic and elderly patients (22). Additionally, 71% of the PanIN 3-containing pancreases showed cystic changes and lower-grade PanIN lesions. Furthermore, in mouse models of PDAC, full-spectrum PanIN has been observed before tumorigenesis (23). The above evidence supports that PanIN is a precancerous lesion of PDAC. This perspective is further supported by tumor recurrence at the surgical margin containing unresectable PanIN 3 lesions (24). Christine et al. performed single-cell sequencing on tumor cells collected through laser cutting microscopy and confirmed the evolutionary relationship between PanIN and PDAC, with a subset of PDAC originating from adjacent PanIN, which provides persuasive evidence that PDAC originates from PanIN (25, 26). Notably, PanIN 1 shows a low rate of progression to high-grade disease, and the probability of progression to invasive cancer from a single PanIN is only 0.86% (27). Therefore, given the prevalence of these asymptomatic low-grade PanIN lesions, distinguishing between PanIN 2 and PanIN 3 is clinically critical.

## 3 Cellular origin of PanIN

**Figure 1:**
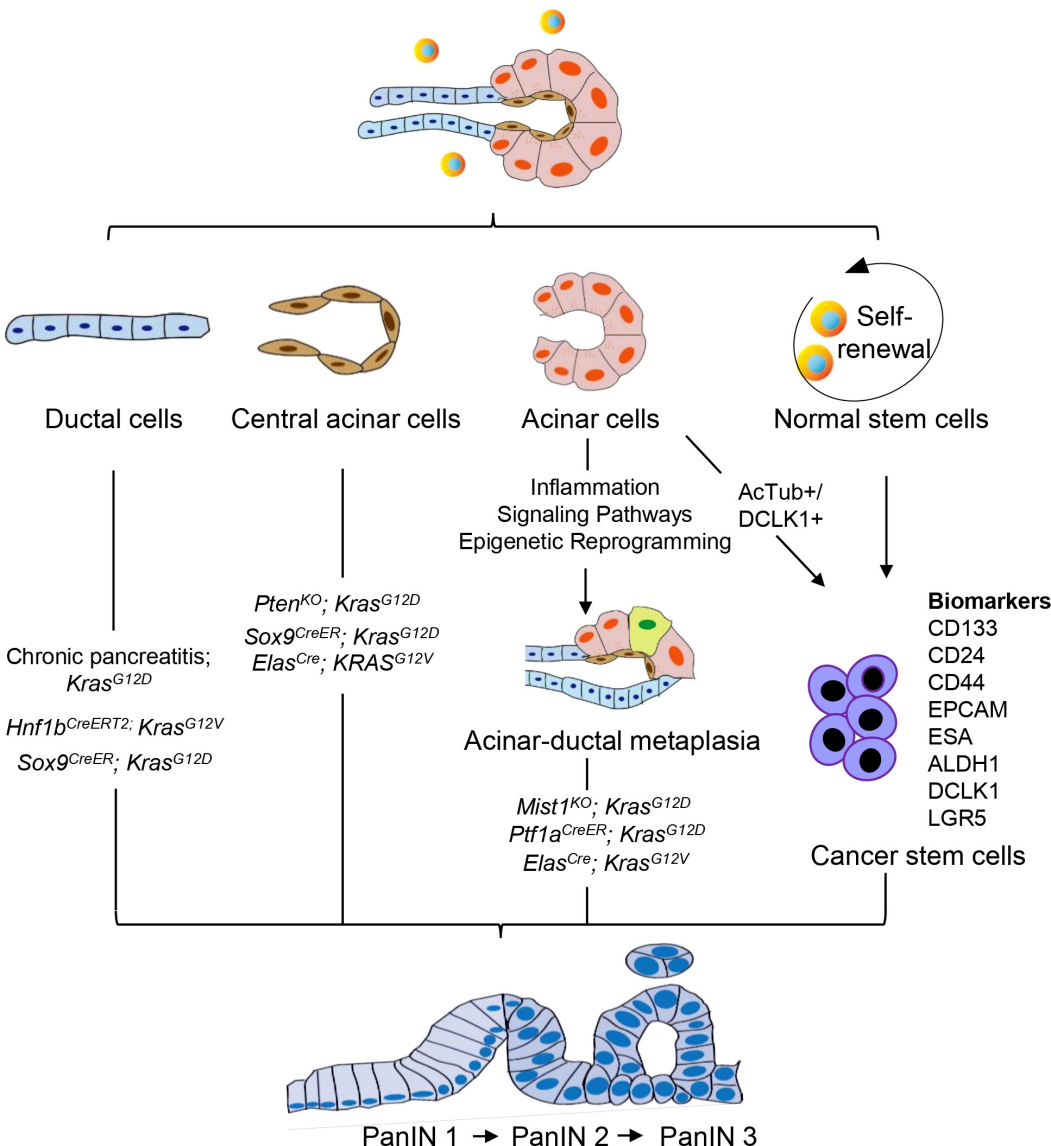
Cellular origin of PanIN. PanIN does not appear to arise *de novo*, but instead derived from the neoplastic transformation of normal cells. PanIN arises from various cell types within the exocrine compartment of the pancreas, including ductal, acinar, and central acinar cells, as well as stem cells. These cell populations serve as the source for the development of PanIN lesions.

The cellular origin of PDAC remains a topic of various hypotheses. The current perspective is that PDAC is a disease localized to the exocrine glands of the pancreas. The exocrine cells of the pancreas mainly include the acinar cells that secrete digestive enzymes, the ductal cells that transport the enzymes to the gut, and the central acinar cells that connect the acinar cells to the ductal cells (28). Therefore, PDAC may originate from acinar cells, ductal cells, or central acinar cells (**Figure 1**). Furthermore, primary cilia are believed to play a significant role in modulating growth factor signaling pathways. In PDAC and PanIN, a key characteristic is the arrest of ciliogenesis. This suggests that primary cilia may also be involved in the progression of PanIN lesions (29).

Despite the classification of tumors based on their histological appearance, morphologic features do not necessarily predict cellular origin. Histological analysis indicates that pancreatic intraepithelial neoplasia (PanIN) may originate from ductal epithelium cells, hence its name (17). Conversely, mouse models of PDAC development suggest that in addition to ductal cells, PanIN may also develop from central acinar cells or acinar cells (30–32). The tumor microenvironment significantly influences the emergence of pancreatic carcinogenesis, facilitating the interconversion of various cell types. Therefore, the cellular origin of PanIN remains a controversial topic to date.

### 3.1 PanIN originates from ductal cells

For a long time, the pancreatic duct has been considered as the cellular origin of PDAC due to its similarity to duct-like morphology (33, 34). However, this hypothesis was challenged when genetic mouse models confirmed that acinar cells may also be a cellular source of PDAC (35–37). A fusion of the cytokeratin 19 promoter (specifically active in pancreatic ductal cells) with mutant KRAS failed to induce PanIN or PDAC, which negates the possibility of a ductal origin of PanIN (38). Nevertheless, recent analysis of somatic variants in human PDAC and precursor lesions has confirmed that PanIN is a disease that can spread through the entire ductal system, suggesting that PanIN may still originate from ductal cells. In patients, multiple discrete PanIN lesions often represent a single neoplasm that has spread along the ductal system (32). Therefore, the hypothesis of ductal cells as the origin of PanIN has been proposed once again. KRAS mutation in pancreatic ducts accelerates PDAC’s progression in a mouse model of chronic obstructive pancreatitis (39). A recent study utilizing tamoxifen-dependent CreERT2 mediated recombination mice (*Hnf1b: CreER^T2^; Kras^G12V^*) suggests that ectopic expression and elevated levels of oncogenic mutant KRAS in pancreatic ducts generate early and late PanIN as well as PDAC (40). However, this doesn’t necessarily mean that only ductal cells are the original cells of PanIN, as more evidence suggests the diversification of its origin cells.

### 3.2 PanIN originates from acinar cells

Acinar cells are a crucial type of pancreatic constituent cells that have been found to be the predominant origin cells for KRAS^G12D^-induced PanINs in various studies. Acinar cells are the main type of pancreatic constituent cells. When oncogenic *Kras* is activated in combination with chronic inflammation, a high-fat diet, or mutations in tumor suppressor genes (SMAD4/DPC4 and P53), adult acinar cells lead to the transformation of PanIN lesions into PDAC (41–43). During embryonic development in mouse models, the pancreatic lineage expresses endogenous KRAS oncogene, which can replicate human PanIN and PDAC in a faithful manner. When endogenous KRAS^G12V^ oncogene is expressed in embryonic acinar/centroacinar cells in mice, they produce PanIN and PDAC. These findings suggest that PDAC may originate from acinar/centroacinar cells or their precursors that differentiate into duct-like cells (30).

Studies have shown that changes in the transcriptional network of acinar cells can affect early events induced by KRAS^G12D^ (35, 37, 44). In mice expressing KRAS^G12D^ but lacking MIST1, an acinar-restricted transcription factor, severe exocrine pancreatic defects occur, and the initiation and progression of PanIN are greatly accelerated (44). Since Mist1 is not expressed in centroacinar cells or duct cells, these findings suggest that altering the acinar Mist1 transcriptional network has a profound effect on the development of PanIN. Kopp et al. conducted a comparison of the propensity of ductal/centroacinar cells and acinar cells to form PanIN in response to oncogenic KRAS. To induce recombination of the KRAS^G12D^ allele in ductal/centroacinar cells or acinar cells, they used *Sox9^CreER^* or *Ptf1a^CreER^* mice, respectively. Their findings showed that all *Ptf1a^CreER^;Kras^G12D^* mice displayed abundant PanIN, in contrast, only 57% of *Sox9^CreER^;Kras^G12D^* mice displayed a small amount of PanIN (45).

Guerra et al. created a PanIN model of *LSL-Kras^G12V^;Elas-tTA/tetO-Cre* mice that allows controlled temporal expression of KRAS^G12V^ oncogene in acinar and central acinar cells. Between 1 and 3 months of age in this mice, acinar-ductal metaplasia was observed. The metaplastic acinar structures comprised highly proliferative cells that included both acinar cells and duct-like cells. The Notch, TGF-β, and EGF signaling pathways are typically maintained at high levels in PDAC, providing cells with growth and survival advantages. Metaplastic acinar structures expressed Notch target genes, and exhibited mosaic expression patterns for ErbB2, and pERK. This expression pattern was similar to that of PanIN, which means that both follow similar molecular pathways. Thus, it is possible that KRAS^G12D^-induced PanIN may originate from acinar cells undergoing acinar-ductal metaplasia (46). TGF-a has been found to induce transdifferentiation of acinar cells into ductal cells, further supporting the notion that acinar cells can develop ductal properties and potentially be the origin of PanIN (47). The ductal origin of PanIN is also possible, although this is more unusual (48).

### 3.3 PanIN originates from central acinar cells

The ductal phenotype of PanIN and other PDAC precursor lesions such as IPMN and MCN, suggests that pancreatic ducts are the origin of pancreatic cancer (49). However, recent evidence emerging from mouse models and lineage tracing studies challenges the notion that the pancreatic duct is the exclusive origin of PDAC. A significant amount of evidence suggests that PDAC may also originate from centroacinar-acinar cells through the ADM process or the expansion of centroacinar cells (28, 30, 37). Centroacinar cells, located at the interface of acinar and terminal duct cells, are considered candidates for pancreatic progenitor cells. Centroacinar cells are much like duct cells, with only subtle differences in the characteristics of the apical membrane. Centroacinar cells are a ductal cell type located at the center of acini, with active Notch signaling, and expression of the endocrine differentiation regulator Sox9 (50). SOX9 is a transcription factor required for the maintenance of the pancreatic progenitor pool and for determining pancreatic endocrine and exocrine cell fates (51–53). Wang et al. discovered that SOX9 was expressed in central acinar cells, ductal epithelial cells, PDAC and its precursor lesions, but was rarely expressed in other pancreatic tumors (54). When co-expressed with oncogenic KRAS, SOX9 mutant mice accelerated the formation of PDAC precursor lesions (45). Pten is expressed in pancreatic ducts, central acinar cells, and pancreatic islets. Stanger et al. discovered that mice with pancreas-specific deletion of Pten exhibit extensive ductal metaplasia with developed PanIN lesions and malignant transformation. They further found that centroacinar cells are highly proliferative before the onset of metaplasia, and this hyperproliferative state exists at an even greater level after metaplastic changes. Ductal metaplasia in Pten mutant mice does not arise from acinar cells, concluding that centroacinar cells are instrumental in initiating the process of metaplasia (28). However, more research is necessary to support the evidence that PanIN can originate from central acinar cells. A subset of central acinar cells exhibit several markers, such as SOX9, HES1, and ALDH1B1, which are known to play significant roles in the development of PDAC and are also associated with cancer stem cells. This has led to the hypothesis that a subset of central acinar cells may function as cancer stem cells.

### 3.4 PanIN originates from cancer stem cells

In human PDAC primary xenografts and pancreatic cancer cell lines, a subpopulation of cells with potent tumor initiation or cancer stem cells (CSCs) has been identified by scholars (55–58). CSCs contribute to PDAC initiation and metastasis, are pluripotent, self-renewing, and tumor-forming, and responsible for resistance to chemotherapy and radiotherapy. CSCs account for less than 1% of all pancreatic cancer cells, and their origin remains uncertain. However, it is currently believed that they may emerge from transformed stem/progenitor cells or terminal cell dedifferentiation (59). The reactivation of embryonic programs and the expression of genes related to self-renewal are common to both stem cells and cancer (60–62). Terminally differentiated cells in the adult pancreas also display a high degree of plasticity. Efforts have been made to transform acinar cells into the endocrine lineage, particularly β cells. Pancreatic acinar cells are prone to dedifferentiate into duct-like cell phenotypes in response to stresses such as injury or inflammation. This evidence well demonstrates the ability of terminal cells to dedifferentiate and retrograde (63–66).

Currently, there is limited research on PDAC CSCs. CD133, CD24, CD44, EPCAM, and ESA are currently considered the most comprehensive biomarkers for pancreatic CSCs (67, 68). The CD133 pancreatic CSCs co-express the CXCR4 receptor play a crucial role in tumor metastasis (58). Animal experiments have confirmed a highly tumorigenic subpopulation of CD44 co-express CD24, EpCAM, and CD133 pancreatic cancer cells that exhibit the biological properties of CSCs, making them often resistant to chemo and radiotherapy (55). In addition to cell surface markers, the cellular molecule ALDH1, which catalyzes the oxidation of intracellular aldehydes and transforms retinol to retinoic acid, has also been identified as a pancreatic ductal adenocarcinoma stem cell marker (67). The ABCG autofluorescent vesicular cell subset in PDAC has potent CSCs properties with distinct tumorigenic potential (69). C-Met is also critical in the biology of pancreatic CSCs (55). The double corticoid-like kinase DCLK1 and the G protein-coupled receptor LGR5 containing leucine-rich repeats are also markers of pancreatic CSCs (68). Additionally, 26S proteasome activity, CD90, and side group (SP) are also included in biomarkers of CSCs (70–73).

Although the majority of studies suggest that PDAC arises from PanIN, relatively few investigations have focused on PanIN CSCs. A subpopulation of cells containing high levels of DCLK1 and acetylated αTubulin (AcTub) are present in mouse ADM and PanIN, these cells display unique sphere-forming capacities similar to CSCs. AcTub+/DCLK1+ cell subpopulation in ADM and PanIN epithelium is acinar cell-derived, and these cells have been shown to enhance clonogenic capacity *in vitro* and have high metastatic potential *in vivo*, both factors are crucial for the development and progression of PanIN (74, 75). Additionally, oncogenic KRAS effectively initiates PanIN development in the DCLK1-expressing cell subset, and deletion of DCLK1 greatly reduces PanIN formation (75). However, it remains to be further clarified and characterized whether these are subpopulations of acinar cells specifically produced by precancerous lesions and PDAC.

## 5 Genetic susceptibility and gene mutation of PanIN

**Figure 2:**
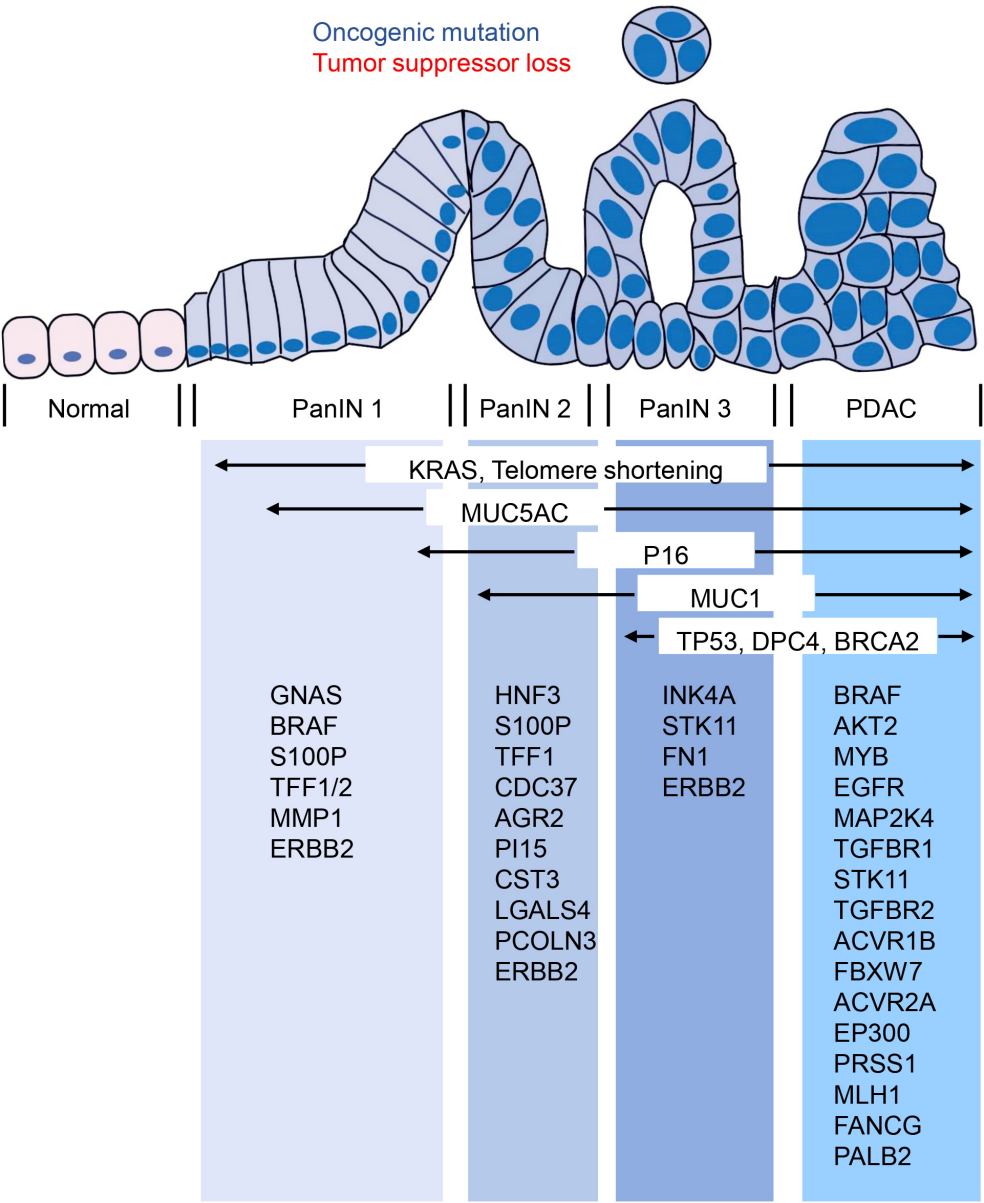
Molecular changes occurring in the progression from PanIN to PDAC. The transition from PanIN to PDAC is often accompanied by several molecular changes that are commonly observed. These include alterations in KRAS, MUC5AC, P16, MUC1, TP53, DPC4, and BRCA2. Furthermore, the figure provided low-frequency molecular mutations that have also been identified during this progression.

Currently, over 90% of invasive PDAC cases are recognized as having KRAS mutations (76, 77). Additionally, the most frequent tumor suppressor genes - CDKN2A, TP53, SMAD4, and BRCA2 - have been found to be inactivated in PDAC (76). Interestingly, CDKN2A acts as an inhibitor of copper oxidation and is inactivated in both PanIN and PDAC. This suggests that copper-iron dysregulation may also occur in PanIN, though this remains unstudied. Whether copper-iron imbalances could serve as diagnostic markers for PanIN, and the potential role of copper metabolism disorders in the progression from PanIN to PDAC, are important questions that still need to be explored. In addition to these, other oncogenes like BRAF, AKT2, MYB, and EGFR, and tumor-suppressor genes including MAP2K4, TGFBR1, STK11, TGFBR2, ACVR1B, FBXW7, ACVR2A, and EP300 can also be mutated in a few PDAC cases (76). Epigenetic changes can also alter gene function in PDAC, and changes in microRNA expression also seem to contribute to cancer development and progression. Since PanIN - the most common precursor lesion of PDAC - is challenging to detect using current imaging techniques, genetic and molecular analysis of PanIN becomes even more crucial. PanIN acquires genetic and epigenetic alterations during its development that allow it to progress into aggressive PDAC (76). Studies have shown that low-grade PanIN increases with age, while high-grade PanIN is present in aggressive PDAC (78). A family history of PDAC poses a significant risk, with patients having a genetic susceptibility to PDAC often exhibiting multiple levels of PanIN in their pancreas (79). Furthermore, individuals with a family history of PDAC are at a greater risk of developing PDAC themselves (80). Several genes that predispose to familial aggregation of PDAC include BRCA2, CDKN2A/p16, STK11/LKB1, PRSS1, MLH1, FANCG, and PALB2 (81–83). Although genetic susceptibility to PDAC can’t be entirely explained by susceptibility gene mutations, by understanding the genetic makeup of PanIN using genetic and molecular analysis, important insights into PDAC can be obtained to facilitate the development and advancement of diagnosis and treatment. Therefore, estimating the evolutionary history of PanIN using genetic and molecular mutations may provide better opportunities for early screening and diagnosis of pancreatic cancer (**Figure 2**).

### 5.1 Gene mutations that occur early in PanIN

According to studies, multiple genetic alterations found in PDAC are also present in PanIN, and their frequency increases with PanIN grade (84). Recently, genomic DNA samples obtained by microdissection from PDAC and related PanIN 2 and PanIN 3 lesions in ten pancreatic cancer patients were subjected to exome sequencing analysis. The results showed that all adjacent lesions originated predominantly from a common ancestral source, with most somatic mutations present at the PanIN 2 stage or earlier (85). Laser microdissection technology combined with pyrosequencing was also used by Michael Goggins et al. to perform more detailed genetic testing of the early development of PanIN. They found that almost all early development of PanIN requires somatic mutation, with KRAS mutation initiating PanIN formation in genetically engineered mouse models (86). More than 99% of PanIN 1 lesions contain KRAS mutation, while early sporadic p16/CDKN2A, GNAS or BRAF mutations may further promote PanIN development based on KRAS mutation (86). Based on the view that PanIN 2 is the first true precancerous stage of PDAC, some scholars have screened 30 potential early diagnostic genes in this stage, including HNF3, S100P, TFF1, CDC37, AGR2, PI15, CST3, LGALS4, and PCOLN3, which have received attention. However, there is much more to PanIN than early genetic and molecular changes that remain to be explored in depth (87).

#### 5.1.1 The earliest genetic changes

Undoubtedly, genetic mutations play a crucial role in the development of PDAC. However, there is controversy surrounding whether the transition from PanIN to PDAC requires the gradual accumulation of mutations. Patients’ mutation loads show great heterogeneity. KRAS oncogene activation and ERBB2 amplification are the earliest genetic changes observed in PanIN (78).

Among PDAC and PanIN, the primary and most important gene under study is KRAS. A meta-analysis showed that the frequency of KRAS mutations in PanIN lesions increased with the lesion grade in PDAC patients, while in those with chronic pancreatitis, the proportion of KRAS-harboring PanIN lesions was relatively low and independent of their grade (84). Recent research confirmed that the oncogene KRAS dose is a key factor in PDAC, with two-thirds of PDAC patients increasing the dose of KRAS^G12D^ (also known as KRAS^iGD^). Researchers found that after the initial KRAS mutation, cancer evolution requires an increased dose of oncogenes by amplifying mutated KRAS or other oncogenes such as YAP1 or NFKB2, through analyses of human PanIN lesions. Different mouse models showed that an increased dose of oncogenes depends on inactivated tumor suppressors. For example, TP53 or CDKN2A homozygous inactivation make tumors prone to KRAS^iGD^, while YAP1 or NFKB2 amplification results from loss of heterozygous CDKN2A. Integrated cell phenotypes revealed that KRAS mutation and KRAS^iGD^ were related to epithelial-tumor mesenchymal transformation and dedifferentiation degrees. Nevertheless, KRAS dose remains an overlooked research area in PDAC and PanIN relative to KRAS mutation. Thomas et al. argue that the carcinogenic dose should be seen as a basic and important process, reminding researchers from a new perspective that KRAS^iGD^ plays a vital role in the formation of PDAC and PanIN (88, 89).

The ERBB2 protein is responsible for encoding the tyrosine kinase growth factor receptor, a key factor in activating and inducing cell proliferation. As a result, ERBB2 is a potent oncogene and its overexpression is an early and significant factor in the development of pancreatic cancer. In fact, research has shown that ERBB2 overexpression is present in 82% of PanIN 1A lesions and 100% of PanIN 3 lesions (90). Moreover, ERBB2 plays a critical role in mediating growth factor-related signal transduction in pancreatic duct lesions, making it a target for potential therapies.

#### 5.1.2 changes in low-frequency mutations associated with senescence

Apart from the well-established driver mutations of PDAC, such as KRAS, TP53, CDKN2A, and SMAD4, there are numerous genes with low-frequency mutations that may play a functional role in promoting tumor development (91). Low-frequency mutations acquired at an early stage could be critical in tumorigenesis. Among the genes with low-frequency mutations in PDAC, ARID1A is one of the most frequently mutated epigenetic regulatory factors in many cancers. A mouse model study analyzing the transcriptome of individual PanIN revealed that *Arid1a* knockout effectively reduced KRAS-induced senescence in PanIN lesions (91). Cellular senescence is a major rate-limiting step in KRAS-driven PanIN progression. Thus, with a significant attenuation of senescence, ARID1A knockout can accelerate PanIN progression (91, 92).

The telomere, a unique structure located at the end of a chromosome, plays a crucial role in maintaining chromosome stability. Severe telomere shortening may result in human chromosome instability, which is necessary for developing most human epithelial cancers (93). Nevertheless, telomere shortening alone is unlikely to induce tumor formation; instead, it sets the foundation for chromosome abnormalities. Notably, telomere length was significantly reduced in 91% of PanIN 1A lesions, indicating that telomere shortening is likely the most common early genetic abnormality in PDAC progression models (93). However, some studies have suggested that this phenomenon may be a result of the activation of the oncogene stress-induced aging process and cannot serve as an initiating factor for PanIN (94).

### 5.2 Activation of cell fate regulatory signal pathway

**Figure 3:**
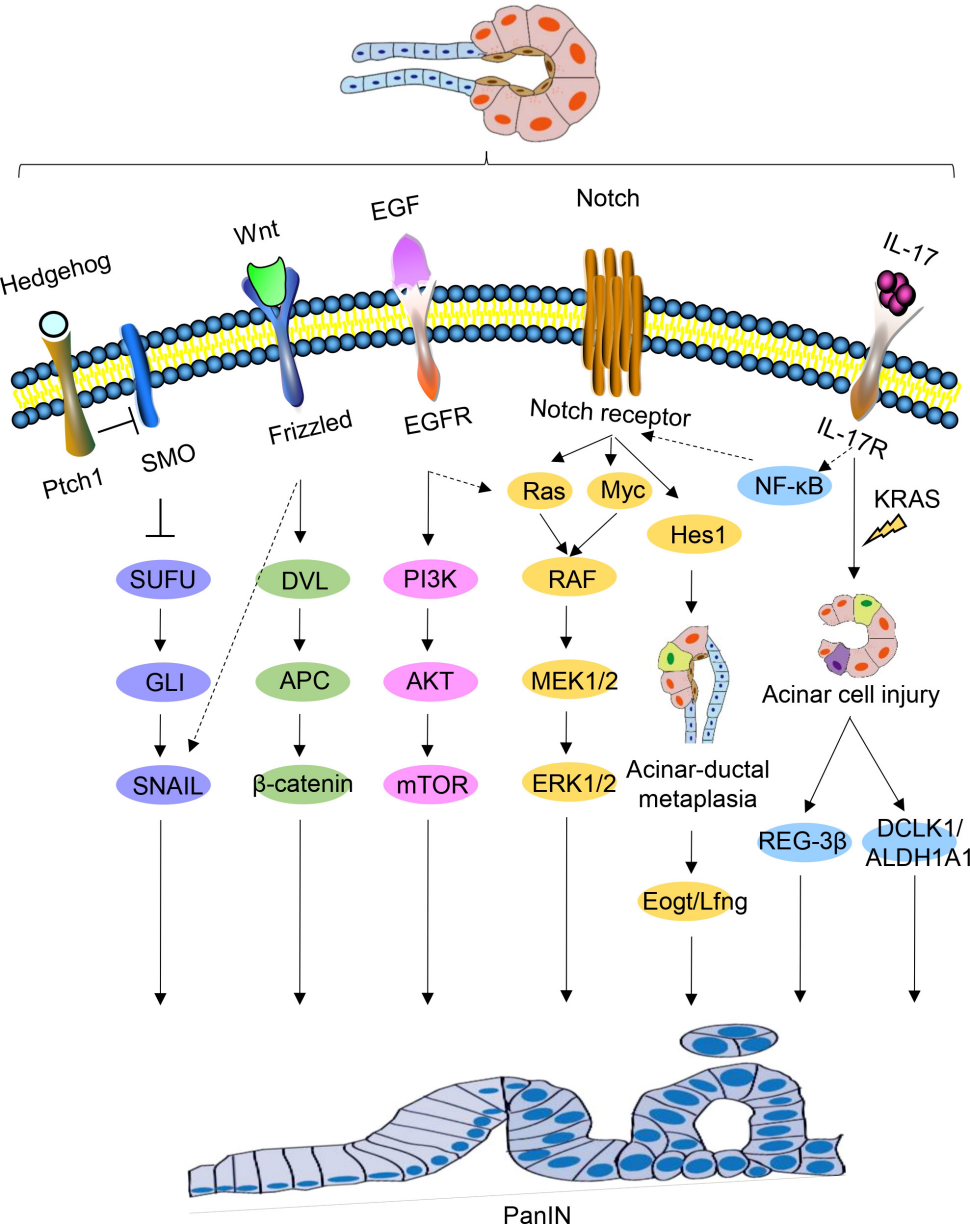
Signaling pathways regulating PanIN. Schematic representations of signaling pathways implicated in PanIN, namely Hedgehog, Wnt, EGF, Notch, and IL-17, are depicted. Certain interactions between these signaling pathways are indicated.

The use of genetically engineered mouse models has significantly advanced the study of PanIN and PDAC. By continuously monitoring the signaling pathways involved in pancreatic carcinogenesis using these models, researchers have been able to gain a better understanding of the mechanisms driving PDAC precursor progression. Activation of numerous cell fate regulation signaling pathways occurs during PanIN and PDAC and plays a crucial role in PDAC development (**Figure 3**).

#### 5.2.1 Activation of Hedgehog pathway

During embryonic life, Hedgehog signaling is critical for pancreatic development, and abnormal activation of this pathway may be a feature of human PanIN (95). Some studies have found that the Hedgehog pathway is abnormally activated in both human PanIN and PDAC (95, 96). Transfection of HPDE cells with Gli1, a downstream mediator of Hedgehog, led to the upregulation of foregut epithelial transcripts (97). Abnormal Hedgehog signaling may contributes to the production of intestinal phenotype in the pancreatic epithelium and increasing neoplastic potential.

#### 5.2.2 Activation of Notch pathway

The Notch signaling pathway plays a key role in cell fate and differentiation decisions, and its early activation in cancer indicates that it plays an important role in cancer initiation and transformation (98). Although the origin of PDAC remains unclear, Notch signaling activation during PanIN initiation is believed to be a key step in PanIN transformation, as activated Notch signaling and KRAS have a synergistic effect on inducing PanIN formation. Notch signaling renders acinar cells sensitive to KRAS^G12D^-driven PanIN initiation, and the coactivation of Notch and KRAS promotes ADM to accelerate the formation of induced PanIN (99).

Notch receptors, NOTCH1 and NOTCH2, are expressed in pancreatic acinar cells and ductal cells, respectively. NOTCH2 deficiency, but not NOTCH1 deficiency, can eliminate PanIN progression, delay the development of PDAC, and prolong survival. NOTCH2 expression is observed during PanIN and PDAC development, and its activation may be necessary for PanIN progression (98). HES1, a key downstream target of the Notch signaling pathway, plays a vital role in acinar cell integrity and cell damage plasticity (100).

Myc amplification supports the early role of PanIN progression in precursor lesions (101). Recently, quantitative proteomic screens identified Myc expression in PanIN 3 lesions, Myc amplification supports its early role in PanIN progression (101, 102). During PanIN progression, Myc expression increases under the regulation of NOTCH2. Furthermore, under active Notch signaling, Myc and Ras signaling cooperate to promote tumor progression. MIST1, a basic helix-loop-helix transcription factor, is only expressed in terminally differentiated pancreatic acinar cells (103). In pancreatic acinar cells lacking MIST1, the EGFR, NOTCH, and KRAS pathways coordinate to accelerate PanIN formation. Shi et al. demonstrated that KRAS^G12D^ mice exhibit severe pancreatic exocrine deficiency in the absence of MIST1, which could be rescued by ectopic expression of Mist1 in acinar cells. The development of PanIN was significantly accelerated in the pancreas of *Mist1KO; Kras^G12D^* mice. *In vitro* studies have shown that *Mist1KO* acinar cells can easily converted to ductal phenotypes and activate EGFR and Notch signal pathways (104).

#### 5.2.3 IL-17

IL-17 is a potent pro-inflammatory cytokine that is closely associated with the formation, growth, and metastasis of a variety of malignancies (105). Activated IL-17 induces DNA damage and pancreatic inflammation, further promoting tumorigenesis in the context of KRAS mutation (106, 107). Notably, there is a positive correlation between IL-17 and NOTCH expression. The IL-17 axis upregulates NOTCH activity through the classical NF-κB pathway, synergistically promoting the production and development of PanIN and PDAC (108).

Moreover, IL-17 has been found to induce the production of pancreatic CSCs (109). In the presence of oncogenic KRAS, IL-17 induces transcription of DCLK1 and ALDH1A1 (a marker of embryonic stem cells) through classical pathway activation, and directly upregulates DCLK1 expression in pancreatic cancer cells in a dose-dependent manner (110). Hence, IL-17 regulates the development of stem cell features of pancreatic cancer cells by increasing the expression of DCLK1, ALDH1A1, and other stem cell markers.

### 5.3 Stemness-related genes

There is a hypothesis that PanIN could potentially arise from CSCs. While the origin of these cells is not entirely clear, there is a shared characteristic between stem cells and CSCs in terms of their expression of genes associated with self-renewal (60, 61). Therefore, alterations in genes related to stemness may signify an early genetic change in the development of PanIN.

#### 5.3.1 SOX9

SOX9 is expressed in multipotent pancreatic progenitor cells and is required for the maintenance of multipotent progenitors in early pancreatic epithelial cells (111). Recent studies have demonstrated that Sox9 is a determinant of ductal cell fate downstream of Notch in the adult pancreas, and is essential for the differentiation and maintenance of ductal cells (112). In the presence of pancreatitis, SOX9 does not contribute to the development of ADM but is critical for its progression into PanIN. The expression of SOX9 is high in low-grade PanIN lesions, while it is comparatively low in high-grade PanINs and PDAC. This pattern suggests that SOX9 plays a critical role in initiating PDAC (113).

#### 5.3.2 ATDC

Ataxia-telangiectasia group D-complementing (ATDC), also known as tripartite motif 29 (TRIM29), is a member of the TRIM protein family, highly expressed in various cancers and associated with prognosis and survival rates (114). In the presence of KRAS^G12D^ expression, ATDC is required for acinar to ductal transdifferentiation in primary acinar cell cultures, as well as in the progression of ADM to PanIN, prompted by cerulein. Additionally, ATDC is required for KRAS^G12D^-induced progression of PanIN and PDAC formation. Therefore, ATDC may play a key role in the reprogramming of pancreatic epithelial cells induced by carcinogenic KRAS.

ATDC activates the β-catenin signaling pathway, leading to the transcriptional activation of SOX9 through TCF4 binding to essential sites in the SOX9 promoter (115). The ATDC-β-catenin-SOX9 signaling axis is critical for the development of pancreatic neoplastic precursors, and in the absence of ATDC, pancreatic neoplastic precursors do not form (116).

#### 5.3.3 DCLK1

DCLK1 is known as a marker of pancreatic progenitor cells and is involved in the development of various gastrointestinal tumors (117). Studies have shown that both mouse and human PanIN and PDAC express DCLK1, and DLK1 may mark tumor-initiating cells in a variety of tumor types (118, 119). In the ApcMin model, pedigree tracing of Cre/lox revealed that cells expressing DCLK1 in intestinal adenoma were responsible for continuous production of tumor cells. Bailey et al. confirmed that DCLK1-positive cells exhibited unique morphology, gene expression patterns, and enhanced “PanIN sphere” formation ability, indicating that DCLK1 may mark a subset of cells with stemness properties in PanIN. However, this requires further confirmation through pedigree tracking (120). Furthermore, pancreas-specific DCLK1 knockout mice showed delayed progression of PanIN lesions (121).

In addition, other factors can interact with DCLK1 and affect the progress of PanIN. G9a, a specific methyltransferase, G9a deficiency attenuates PanIN progression in *LSL-Kras^G12D^;Pdx1-Cre* (KC) mice and prolongs the survival time. Further studies have found that G9a plays an important role in the malignant transformation of low-grade PanIN (122). G9a deficiency reduced the number of DCLK1-positive cells and ERK phosphorylation in PanIN lesions, but the mechanism is unclear. Several studies suggest that G9a may be associated with MAPK activation and the clonogenic capacity of DCLK1-positive cells in the pancreas of KC mice. The direct binding of Dclk1 to KRAS protein is the key factor in the activation of MAPK in pancreatic cancer cells (121), indicating the functional interaction of G9a, DCLK1, and MAPK pathways in KRAS-driven PDAC (122).

### 5.4 Gene mutation in the late stage of PanIN

In the early stages, PanIN’s molecular characteristics are relatively simple, and the likelihood of developing invasive PDAC is low. However, high-grade PanIN (PanIN 3) is more uncertain and complex due to severe nuclear atypia, lumen necrosis, and marked epithelial sprouting, which indicates the development of PDAC (123). At present, high-grade PanIN is not only the main precursor lesion of PDAC but also an ideal target for early detection (124). Unfortunately, clinical and imaging methods cannot detect high-grade PanIN, and thus molecular detection methods are critical (125, 126).

Maitra et al. utilized immunohistochemistry to examine the protein expression of genes related to PDAC progression in 55 PanIN lesion tissue microarrays. Their findings revealed that molecular abnormalities in PanIN were not random and were typically classified as “early” changes (such as prostate stem antigen and MUC5 expression, or P16 loss), “intermediate” changes (such as cyclin D1 expression), and “late” changes (such as P53, proliferating antigen, and MUC1 expression, or Smad4/Dpc4 loss) (127). However, further experiments involving sequencing and pedigree tracking have raised questions about these results. Using tumor sequencing, adjacent PanIN lesions, and normal tissues from 10 PDAC patients, Murphy et al. discovered that the tumors were genetically identical to the adjacent PanIN lesions for over 50% of the genetic mutations. While there were some variations in molecular mutations in each sample, the overall results indicated that the adjacent PanIN and tumor originated from a common ancestor and that most of the somatic mutations in the tumor occurred in PanIN 2 or earlier. Furthermore, PanIN 2 contains as many mutations as PanIN 3 and tumor tissue, indicating that PanIN 2 can potentially progress into a tumor even in the absence of PanIN 3. In addition, precancerous lesions may require epigenetic modifications, aneuploidy, or changes based on the expression of certain proteins to develop into invasive tumors (85). For instance, the proteins gal-1, annexins A4 and A5, ANXA4, vimentin, and laminin, which exhibit differential expression in PanIN 3, are also dysregulated in PDAC (102, 128). C-myc was identified as an important regulatory protein in the dysregulated protein network in PanIN 3 tissue, which supports the pathological and genomic progression model of PanIN to PDAC from a proteomic perspective (129–131).

Hosoda et al. conducted an analysis of 17 high-grade PanIN lesions in 15 patients and 16 low-grade PanIN lesions in 10 patients, revealing a presence of KRAS oncogenic mutations in 94% of both high and low-grade PanIN lesions. Meanwhile, in high-grade PanIN lesions, 29% exhibited RNF43 mutations, 18% displayed CDKN2A mutations, and 12% had mutations in GNAS and TP53. Additionally, one high-grade PanIN lesion was found to have mutations in PIK3CA, TGFBR2, and ARID1A (124). Though mutations in RNF43 and GNAS were also observed in low-grade PanIN lesions, no mutations in CDKN2A or TP53 were detected.

In the development of non-invasive pancreatic duct lesions, the inactivation of the P16 gene has been found to be a contributing factor (132). Analysis via immunohistochemistry of lesional tissues from 70 patients with pancreatic diseases demonstrated that loss of P16 expression occurred in pancreatic tissue with pancreatic duct dysplasia, preceding P53 and DPC4 inactivation. P16 expression inversely governs the expression of P21 and P53, both of which play an important role in cell cycle regulation and the onset and development of PanIN. As such, assessments based on immunohistochemistry can enhance the efficiency of PanIN diagnoses (133–135). While the early identification of high-grade PanIN may facilitate timely treatment, studying PanIN 2 may provide greater clinical diagnostic value and impede the progression of PanIN 2 into high-grade PanIN and aggressive PDAC.

## 6 Diagnosis of PanIN

**Figure 4:**
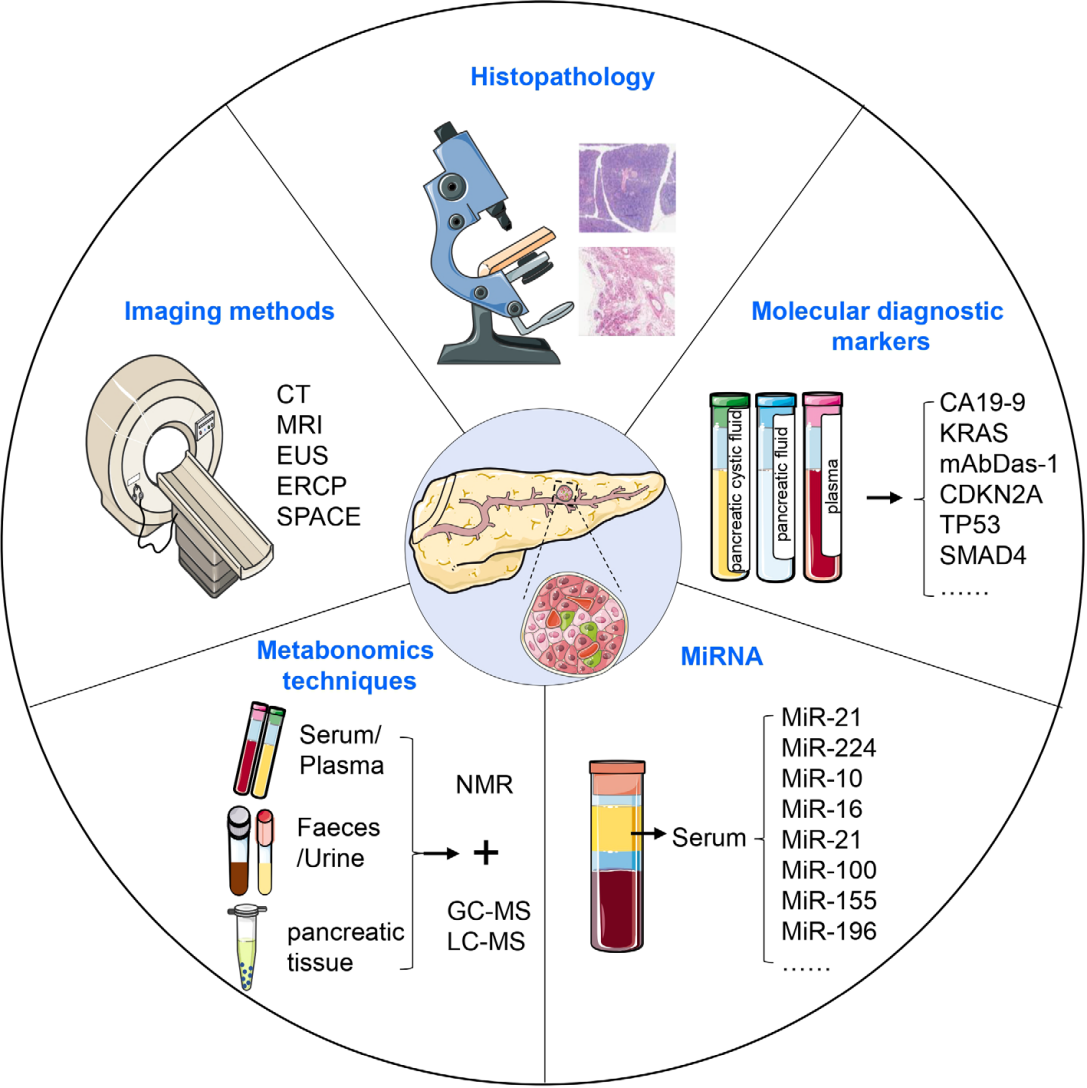
Diagnosis of PanIN. PanIN can be diagnosed using various approaches, including histopathological methods, diagnostic imaging, serum tumor marker detection, metabonomics techniques, and the assessment of aberrant miRNA expression. These diagnostic modalities collectively contribute to the accurate identification and characterization of PanIN lesions.

Research has shown that almost 90% of PDAC is diagnosed after spreading beyond the pancreas, more than 50% of PDAC patients have systemic metastases, and only 15-20% of patients are eligible for surgical resection upon diagnosis (136, 137). Early detection of invasive cancer improves prognosis, but the most significant advancement in survival could come from identifying pre-malignant tumors, which elevates the importance of PanIN to the top of early diagnosis (138). In the WHO classification, carcinoma *in situ* corresponds to high-grade PanIN (PanIN 3) without invasive carcinoma. Carcinoma *in situ* is neither metastatic nor invasive, and it causes some abnormalities that are considered secondary clinical manifestations. PanIN can be diagnosed through histopathological methods, diagnostic imaging such as computed tomography (CT) and magnetic resonance imaging (MRI), serum tumor marker detection, gene diagnosis, circulating tumor DNA biopsy, and circulating tumor cell testing (**Figure 4**) (139–141).

### 6.1 Diagnosis using imaging methods

Histopathology is considered the most accurate and reliable gold standard among all cancer diagnostic methods. However, obtaining sufficient material for biopsy can be challenging, and the invasive nature of tissue collection can affect diagnostic accuracy. The sensitivity of CT and MRI is limited by the size of the tumor. Spiral CT has a sensitivity of only 72% in detecting small pancreatic masses, and MRI performs similarly (142, 143).

Endoscopic ultrasonography (EUS) has been found to have higher diagnostic efficiency than CT and MRI, and EUS-guided fine needle aspiration (EUS-FNA) is particularly superior to histopathological diagnosis. However, EUS is an invasive procedure with potential complications like bleeding and pancreatitis. Endoscopic retrograde cholangiopancreatography (ERCP) is another option to diagnose PanIN, but it carries a postoperative risk of inducing pancreatitis and requires strict candidate selection (144).

Recently, serial pancreatic-juice aspiration cytologic examination (SPACE) has emerged as an advanced method for malignancy diagnosis (145–149). Nakahodo et al. retrospectively compared various imaging methods in patients with high-grade PanIN undergoing surgery and found that focal pancreatic parenchymal atrophy on CT/MRI and a hypoechoic stenotic pattern on EUS are important for the early diagnosis of PDAC, especially in the diagnosis of high-grade PanIN (150, 151). A comprehensive approach should be utilized to improve the diagnosis of PanIN, given the limitations of current imaging techniques and diagnostic methods.

### 6.2 Molecular diagnostic markers

Currently, early detection strategies for PDAC are primarily focused on pancreatic cystic fluid, pancreatic fluid, and plasma samples, but development of universal methods for early detection of PDAC is still necessary. The sensitivity, specificity, and accuracy of tumor marker CA19-9 in the diagnosis of PDAC are 83.1%, 73%, and 75%, respectively, but it is not suitable for early detection of PDAC due to its low value in predicting early stages of PDAC (152, 153). Genetic testing can provide a more sensitive diagnostic approach than conventional methods. Detection of KRAS mutations in the patient’s pancreatic fluid or plasma can offer stronger evidence for the diagnosis of PDAC, although its presence alone is not sufficient for such diagnosis (154). In addition, the protein encoded by GPC in cancer exosomes can be used as a potential marker for non-invasive diagnosis and screening tools by ctDNA biopsy diagnosis (141).

At present, there are few specific biomarkers can distinguish between low-grade PanIN, high-grade PanIN, and PDAC directly. MAbDas-1, a monoclonal antibody against the colonic epithelial antigen, has shown promise as a highly specific marker for precancerous lesions located in the upper gastrointestinal tract. Moreover, MAbDas-1 is capable of distinguishing between low- and high-risk IPMN lesions (155). Through the study of cystic fluid from 169 patients with pancreatic cystic lesions, Das et al. found that the Das-1 ELISA exhibited a sensitivity of 88%, specificity of 99%, and accuracy of 95% for high-risk lesions (156). Expression of Das-1 was not identified in 56 normal pancreatic ducts or 95 low-grade PanIN lesions; however, it was markedly raised in high-grade PanIN and PDAC. As such, mAbDas-1 proves highly specific for advanced PanIN and PDAC and could aid in preoperative diagnoses, as well as clinical risk stratification (157).

Using a PDAC genetically engineered mouse model, Dieter et al. employed cathepsin-activatable near-infrared probes combined with flexible confocal fluorescence laser microscopy to differentiate normal pancreatic tissue, low-grade PanIN, high-grade PanIN, and early PDAC (158). This method offers a promising approach to early diagnosis of PDAC with greater accuracy compared to traditional diagnostic methods.

### 6.3 Metabonomics techniques

Metabolomics techniques offer a promising approach for the detection and identification of cancer by facilitating the comprehensive analysis of trace metabolites in biological fluids and tissues (159–161). Notably, the metabolomic profiles of PDAC patients are distinct from that of healthy controls (161–163). Potential metabolic signatures can be identified by using nuclear magnetic resonance (NMR) spectroscopy combined with multivariate and univariate statistical analysis and screening for differential metabolites including glucose, amino acids, carboxylic acids, and coenzymes (164, 165). However, due to its relatively low sensitivity, NMR should be combined with other techniques like GC-MS and LC-MS to improve biomarker identification for early clinical detection and diagnosis of PDAC (165, 166). By effectively combining these techniques, researchers can maximize the potential of metabolomics as a diagnostic tool for PDAC.

### 6.4 MiRNA

MicroRNAs (miRNAs) are a class of small non-coding RNAs that are closely associated with cancer progression (167). Abnormal expression of miRNAs is believed to be an early event in pancreatic carcinogenesis (168). MiRNAs are implicated not only in PDAC progression but also in the process from PanIN to cancer. MiR-21 activates KRAS signaling via the transcription factor ELK1, promoting tumor cell proliferation, migration, and invasion (169). MiR-224 overexpression activates normal fibroblasts into tumor-associated fibroblasts, thereby increasing drug resistance in tumor cells (170). These studies demonstrate the significant impact of miRNA on promoting disease progression. Moreover, miRNAs are abundant and stable in serum, making them a promising marker for clinical diagnosis of PDAC (171–173). Several studies have identified specific miRNAs in PanIN, including miR-10, miR-16, miR-21, miR-100, and miR-155, which are not found in normal pancreatic ducts (174). Additionally, miR-196b has been identified as a potential biomarker for differentiating PanIN 3 from low-grade PanIN, thus facilitating screening of high-risk cancerous lesions and assisting in clinical treatment selection.

## Conclusion

PanIN can arise from multiple cell types and progress to PDAC through various signaling pathways driven by genetic mutations. Typically, this process involves a transition from low-grade to high-grade PanIN, which presents an opportunity to block invasive cancers. Although PanIN is recognized as a precursor lesion, there is often a greater focus on diagnosing PDAC, indicating a lack of understanding of PanIN. By utilizing genetic testing and non-invasive biomarkers to diagnose PanIN and determine its grade, early intervention can be achieved, leading to improved patient outcomes. Therefore, it is critical to conduct in-depth research into the mechanisms underlying PanIN, as this has significant clinical implications. The current challenge is to accurately grade PanIN lesions and develop effective treatment strategies.

Preventing the progression of PanIN2 to PanIN3 and the subsequent transition from PanIN3 to PDAC is crucial, yet no established treatment exists. High-risk patients are typically advised to adopt healthier lifestyle choices, such as quitting smoking, maintaining a balanced diet, and engaging in regular physical activity. Some studies suggest that non-steroidal anti-inflammatory drugs, metformin, and other agents may help reduce the risk of progression from PanIN to PDAC. Additionally, in cases of high-grade PanIN with a significant risk of progression, surgical resection may be considered. In conclusion, the management of PanIN remains an evolving field, with treatment strategies likely to evolve as new research emerges.
